# Efficacy of vedolizumab as maintenance therapy in a patient with ulcerative colitis on hemodialysis for end-stage renal failure: a case report

**DOI:** 10.3389/fmed.2025.1616425

**Published:** 2025-09-25

**Authors:** Zezheng Wang, Jing Tang, Shiying Wang, Haiyan Zhang

**Affiliations:** ^1^The Second School of Clinical Medicine, Guangzhou University of Chinese Medicine, Guangzhou, China; ^2^Department of Gastroenterology, Guangdong Provincial Hospital of Traditional Chinese Medicine, Guangzhou, China; ^3^Guangdong Provincial Key Laboratory of Clinical Research on Traditional Chinese Medicine Syndrome, Guangzhou, China

**Keywords:** vedolizumab, hemodialysis, ulcerative colitis, end-stage renal failure, case report

## Abstract

**Background:**

Hemodialysis is an important life-sustaining treatment for patients with end-stage renal failure (ESRF), but its ultrafiltration process may induce hypoalbuminemia and compromise systemic immune competence. In Ulcerative colitis (UC), immune dysregulation underlies chronic inflammation. Vedolizumab (VDZ), a humanized monoclonal antibody selectively targeting the α4β7 integrin expressed on gut-homing lymphocytes, demonstrates efficacy in moderate-to-severe UC. However, its pharmacokinetic profile in hemodialysis-dependent patients remains poorly defined due to limited data in this population.

**Case presentation:**

A 50-year-old male with ESRF secondary to renal allograft failure (2012) received maintenance thrice-weekly hemodialysis. In February 2022, he developed mucoid and bloody purulent stools and was diagnosed with UC. Initial therapy with oral mesalazine (4 g daily) failed to control symptoms, resulting in clinical deterioration. Subsequently, VDZ was initiated at a standard induction regimen: 300 mg intravenous infusions at weeks 0, 2, 6, followed by maintenance dosing every 8 weeks.

**Intervention and outcome:**

Clinical remission with complete mucosal healing (Mayo endoscopic score 0) was achieved by week 22. Post-dialysis trough serum VDZ concentration measured 13.35 μg/mL, with no detectable anti-drug antibodies. Renal function remained stable throughout (estimated glomerular filtration rate [eGFR] 3.45 mL/min/1.73m^2^), confirming therapeutic efficacy without exacerbation of uraemic complications.

## 1 Introduction

End-stage renal failure (ESRF), defined as irreversible cessation of renal function secondary to advanced chronic kidney disease (CKD), disrupts critical physiological processes including glomerular filtration and tubular reabsorption, necessitating renal replacement therapy. Hemodialysis remains the cornerstone of management for ESRF patients not eligible for transplantation, yet its extracorporeal ultrafiltration imposes significant metabolic and immunologic burdens (1). Notably, hemodialysis-induced protein losses may exacerbate hypoalbuminemia and compromise systemic immune surveillance, contributing to a proinflammatory milieu that complicates comorbid inflammatory disorders (2).

In ulcerative colitis (UC), immune dysregulation—specifically dysregulated lymphocyte trafficking to the gastrointestinal mucosa—is a critical pathological mechanism (3). This mechanistic insight underscores the therapeutic utility of biologics targeting leukocyte migration, with vedolizumab (VDZ) emerging as a gut-selective monoclonal IgG1 antibody directed against the α4β7 integrin. By inhibiting lymphocyte homing to the intestinal mucosa, VDZ restores mucosal immune homeostasis while minimizing systemic immunosuppression, rendering it a preferred option for moderate-to-severe UC (4).

Despite VDZ’s established efficacy in UC, its pharmacokinetic profile in hemodialysis-dependent patients remains underexplored. Hemodialysis-associated ultrafiltration may theoretically alter drug clearance and immunogenicity, yet real-world data addressing these variables in ESRF cohorts are scarce. Here, we describe an illustrative case of a hemodialysis-dependent ESRF patient with refractory UC achieving clinical remission following VDZ initiation, highlighting the need for further investigation into the drug’s performance in this unique population.

## 2 Case report

A 50-year-old male with a history of renal transplantation in 2012 (maintained on chronic immunosuppressive therapy) developed end-stage renal failure (ESRF) by 2020, requiring thrice-weekly hemodialysis.

The patient’s symptoms began in February 2022, with recurrent episodes of mucoid and bloody purulent stools. An initial diagnosis of UC was made at an external institution at that time, and oral mesalazine (2 g twice daily) was prescribed. However, the patient did not seek further standardized medical care and was lost to regular follow-up for approximately 1 year. During this interval, he reported persistent symptoms and worsening disease activity.

**Figure 1:**
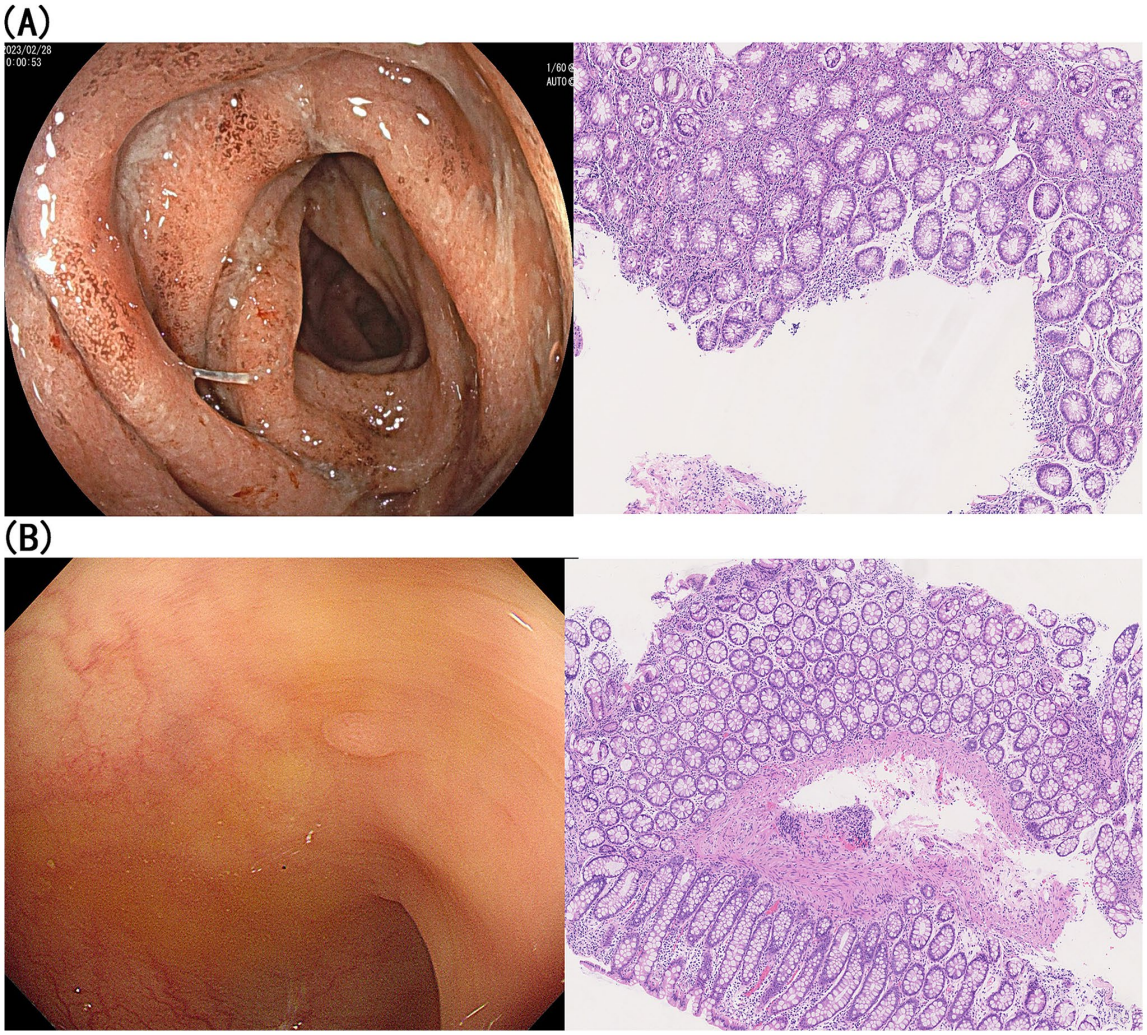
The colonoscopy and histopathological examination of sigmoid colon. **(A)** Colonoscopy and histopathology conducted in February, 2023; **(B)** colonoscopy and histopathology conducted in August, 2023.

In February 2023, he was referred to our institution with a chief complaint of frequent (5–6 per day) mucoid and bloody purulent stools, abdominal pain, and tenesmus. Given his immunosuppressed state and an elevated serum procalcitonin (1.45 ng/mL), a thorough infectious workup was performed to rule out concurrent pathologies before confirming the UC flare. Crucially, stool analysis revealed a positive *C. difficile* GDH antigen but a negative toxin A/B assay, consistent with asymptomatic colonization rather than active infection. Further stool tests, including bacterial and fungal cultures, rotavirus antigen, and interferon-gamma release assay (IGRA), were all negative. Based on these findings, an active infectious colitis was ruled out, and antimicrobial therapy was not indicated (Figure 1).

Following a comprehensive reevaluation, the diagnosis of chronic relapsing, left-sided, and active moderate stage ulcerative colitis (UC) coexisting with ESRF was confirmed. Despite initiation of oral mesalazine (2 g twice daily), the patient’s symptoms of mucoid and bloody stools persisted, and his clinical condition deteriorated, underscoring inadequate disease control and the need for advanced therapeutic strategies.

Following exclusion of contraindications, the patient was initiated on vedolizumab (VDZ) therapy, administered as 300 mg intravenous infusions on March 2, March 16, April 13, June 8, and August 3, 2023 (total of 5 doses). Hemodialysis for ESRF was maintained without complications throughout this period. A comprehensive follow-up evaluation was performed before the patient’s fifth VDZ infusion. The patient’s key clinical characteristics are detailed in Table 1.

**Table 1:** Clinical characteristics of the patient at diagnosis and after vedolizumab therapy.

Variable	At diagnosis (Feb 2023)	After induction therapy (Aug 2023)
Laboratory findings		
Erythrocyte sedimentation rate (mm/h)	32	38
Procalcitonin (ng/mL)	1.45	1.51
Creatinine (μmol/L)	1,373	1,286
Serum albumin (g/L)	42.9	44.1
Urea (mmol/L)	34.73	30.33
eGFR (mL/min/1.73m^2^)	3.19	3.45
Hemoglobin (g/L)	135	138
C-reactive protein (mg/L)	2.65	5.71
EBV VCA-IgG (U/mL)	703.00	742.00
EBV NA-Ig (U/mL)	430.00	462.00
*C. difficile* GDH antigen	Positive	Positive
*C. difficile* toxin A/B	Negative	Negative
Interferon-Gamma Release Assay (IGRA)	Negative	Negative
Anti-CMV IgM (COI^a^)	0.19	0.21
EBV-DNA (copies/mL)	Positive (7.64 × 10^3^)	Negative (< 5.0 × 10^2^)
CMV-DNA (copies/mL)	Negative (< 5.0 × 10^2^)	Negative (< 5.0 × 10^2^)
Stool rotavirus antigen	Negative	Negative
Stool bacterial culture	Negative	Negative
Stool fungal culture	Negative	Negative
Stool routine analysis	Microscopy: Unremarkable; Occult Blood: Positive (2+)	Microscopy: Unremarkable; Occult Blood: Negative (−)
Vedolizumab concentration (μg/mL)	Not administered	13.35^b^
Anti-vedolizumab antibodies	Not administered	Negative^b^
Endoscopic findings		
Mayo endoscopic score	2	0
Description	(10–25 cm from the anal verge) Loss of vascular pattern; fragile, granular mucosa with contact bleeding; patchy erosions with purulent exudate; continuous lesions; no pseudopolyps; remaining colorectal mucosa appeared smooth without bleeding, ulcers, or masses.	No significant abnormalities were observed in the ileocecal valve, ascending, transverse, descending, sigmoid colon, or rectum; mucosal surface smooth with clear vascular pattern; no ulcers identified.
Histopathological findings		
Site	Sigmoid colon	Sigmoid colon
Description	Mucosal erosion; relatively preserved crypt architecture; moderate inflammatory infiltrate (neutrophils, plasma cells, lymphocytes); focal lymphoid hyperplasia with follicle formation; no crypt abscesses or granulomas identified.	Mucosal erosion; uniform crypt distribution; moderate inflammatory infiltrate (neutrophils, plasma cells, lymphocytes); focal lymphoid hyperplasia with follicle formation; no crypt abscesses or granulomas identified.

eGFR, estimated glomerular filtration rate; EBV, Epstein–Barr virus; VCA-IgG, viral capsid antigen immunoglobulin G; NA-Ig, nuclear antigen immunoglobulin G; GDH, glutamate dehydrogenase.

𝐼OI, Cutoff Index. A value ≥ 1.0 is considered positive.

ᵇMeasured 1 h after hemodialysis.

The VDZ results confirmed adequate drug exposure without immunogenicity. The resolution of symptoms (including mucoid bloody stool, abdominal pain, and diarrhea) combined with endoscopic evidence indicates that the patient achieved clinical remission and mucosal healing. The patient continues on maintenance therapy with VDZ infusions and oral mesalazine (2 g once daily), alongside thrice-weekly hemodialysis. He remains clinically stable with no adverse events reported, achieving sustained remission of UC and optimized renal replacement therapy.

## 3 Discussion

Vedolizumab (VDZ), a clinically validated biologic for moderate-to-severe ulcerative colitis (UC), has redefined treatment paradigms through its dual capacity for rapid remission induction and long-term maintenance. Our case is unique, demonstrating the successful use of VDZ in a patient with UC on maintenance hemodialysis for end-stage renal failure (ESRF), a context with limited clinical data. Crucially, this case highlights a significant clinical paradox: the onset of UC in a patient with a history of renal transplantation and long-term systemic immunosuppressive therapy. This discussion will explore the potential role of prior immunosuppression in the pathogenesis of what can be termed “secondary IBD” (5), and the subsequent efficacy of gut-selective therapy in this complex setting.

The development of UC in this patient, despite years of systemic immunosuppression, aligns with the recognized phenomenon of *de novo* IBD in solid organ transplant (SOT) recipients. The incidence of *de novo* IBD in the SOT population is significantly higher than in the general population, estimated to be up to ten-fold greater (6). This paradoxical effect is thought to arise from the dysregulation of the intestinal immune system induced by the immunosuppressive therapy itself (7, 8). Immunomodulators commonly used in transplantation, such as tacrolimus and mycophenolate mofetil (MMF), may down-regulate regulatory T-cells in the colonic mucosa (5). These cells are critical for preventing abnormal immune responses against commensal microflora. Their impairment creates a propensity for immune-mediated inflammation, allowing for a continuous and inappropriate immune response to intestinal antigens that culminates in colitis. Indeed, both tacrolimus and MMF have been specifically associated with an increased risk of *de novo* IBD development post-transplantation (5, 6). In our patient, the late onset of UC after years of chronic exposure to immunosuppressants is consistent with these reports, which suggest a key etiopathogenetic role of chronic immunosuppression (7).

The efficacy of VDZ in this complex setting underscores the pivotal role of its gut-selective mechanism. The pathogenesis of the patient’s UC suggests that his systemic immunosuppression was insufficient to control the specific pathways of mucosal lymphocyte homing that drive colitis. Vedolizumab, by specifically targeting the α4β7 integrin, precisely inhibits this pathway, allowing for potent local inflammation control at the colonic mucosa while avoiding further compounding the patient’s systemic immunosuppression (8). This targeted approach is particularly advantageous in SOT recipients. A recent systematic review and meta-analysis on the use of biologics in SOT recipients found that such therapies are generally well-tolerated and do not appear to increase the risk of infectious complications compared to the general SOT population (9). Specifically for vedolizumab, its intestinal selectivity is thought to reduce the adverse effects of systemic immunosuppression, contributing to its high efficacy and excellent tolerability in this population (8). The successful achievement of clinical and endoscopic remission in our patient, without reported infectious complications, supports vedolizumab as a safe and effective therapeutic option for managing *de novo* IBD in immunosuppressed transplant recipients.

Stable VDZ pharmacokinetics are pivotal for maintaining therapeutic efficacy. While the patient’s initial serum procalcitonin (PCT) was elevated at 1.45 ng/mL, a level suggestive of bacterial infection, its interpretation requires caution in this specific patient population. Extensive literature indicates that patients with end-stage renal failure (ESRF) on hemodialysis often exhibit basally elevated PCT levels in the absence of infection, due to reduced renal clearance and a chronic inflammatory state associated with uremia (10–13). For instance, studies have reported mean PCT concentrations in non-infected hemodialysis patients to be as high as 0.69 ng/mL to 2.00 ng/mL (11, 12, 14). Consequently, a higher diagnostic cutoff is often recommended, with several investigators suggesting a threshold of ≥1.5 ng/mL should be used to diagnose severe infection in this population (10–12).

As detailed in our comprehensive infection workup, an active bacterial colitis was definitively ruled out. Another concern in ESRF patients is that altered physiological states, such as hypoalbuminemia, might accelerate drug clearance (15). However, VDZ’s high molecular weight (147 kDa) precludes its clearance via conventional hemodialysis membranes, as evidenced by the stable post-dialysis trough concentration (13.35 μg/mL) observed here. This indicates that adequate drug exposure was maintained despite regular hemodialysis. Furthermore, the absence of anti-drug antibodies (ADAs) suggests that the patient’s underlying immune status did not increase the risk of immunogenicity, reinforcing the validity of VDZ as a sustained intervention in this case.

## 4 Conclusion

This case illustrates the paradoxical development of *de novo* ulcerative colitis in an ESRF patient with a history of renal transplantation and chronic immunosuppression. The sustained therapeutic response observed here supports vedolizumab’s retained efficacy and favorable tolerability profile in this complex patient subset. Our experience highlights that a gut-selective biologic therapy can be a feasible and safe strategy, effectively managing localized intestinal inflammation without adding to the burden of systemic immunosuppression. Given the demographic shift toward an aging UC population and emerging evidence linking immune dysregulation to ESRF progression, there exists a critical need for large-scale, prospective clinical trials to further investigate vedolizumab’s performance in this unique population.

## Data Availability

The original contributions presented in the study are included in the article/Supplementary material, further inquiries can be directed to the corresponding author.
